# Policies for Social and Health Equity: The Case for Equity Sensitive Universalism: Comment on "Implementing Universal and Targeted Policies for Health Equity: Lessons From Australia"

**DOI:** 10.34172/ijhpm.2022.7573

**Published:** 2022-11-06

**Authors:** Rebecca Mead, Chrissie Pickin, Jennie Popay

**Affiliations:** ^1^Division of Health Research, Faculty of Health and Medicine, Lancaster University, Lancaster, UK.; ^2^Retired from Department of Health and Human Services, Melbourne, VIC, Australia.

**Keywords:** Universalism, Targeting, Social Dividend, Health Equity, Stigma

## Abstract

This commentary reflects on an important article by Fisher and colleagues who draw on four Australian policy case studies to examine how universal and targeted approaches or a combination can be deployed to improve health equity. They conclude that universal approaches are central to action to increase health equity, but that targeting can improve equity of access in some situations including in the context of proportionate universalism. However, we argue that although target services may provide benefits for some populations, they are often stigmatizing and fail to reach may people they aim to support. Instead of accepting the dominant discourse about the key role for targeted approaches, we argue that those committed to reduce social and health inequities should consider the potential of Equity Sensitive Universalism (ESU). This approach focuses on achieving proportionate outcomes with equally provided resources rather than proportionate inputs and provides a ‘cohesion dividend,’ increasing social solidarity.

 In their recent book on the pandemic Bambra and colleagues^1^ argue the world is in the grip of a syndemic, described by Singer^2^ as, “a set of closely intertwined and mutually enhancing health problems that significantly affect the overall health status of a population within the context of a perpetuating configuration of noxious social conditions.” Today these *noxious conditions* include a worsening climate/environmental crisis, the on-going pandemic, rapidly deteriorating economic conditions, plus wars and armed conflicts, all combining to drive ever widening structural and health inequalities. Against this backdrop, the article by Fisher and colleagues^3^ provides timely evidence how positive impacts on health equity from universal and targeted interventions can be maximized.

 Over the past few decades, the balance between universal and targeted approaches to reduce social and health inequalities has shifted. Universal health coverage‎ is recognized as essential for progress on Sustainable Development Goal 3 for health and remains an aspirational goal for many countries. But primary care universalism is an exception. Since the 1980s, neoliberal ideologies, dictating a reduced role for ‘the state’ and a ‘mixed economy’ of welfare, compounded since 2008 by the economic ‘crisis,’ have been shaping global and national policies. National governments have moved at differing speeds to strip away universal services (in health, education, housing, income support, environmental planning and regulation, etc), and the progressive tax systems that supported these. Instead, more targeted provision has been introduced, provided by private and civil society organisations, with responsibility for health and welfare moving to the local state and communities themselves.

 Responding to this ‘reality’ and evidence that universal approaches sometimes failed adequately to address social and health inequalities, the 2008 World Health Organization (WHO) Commission on the Social Determinants of health recommended a mix of universal and targeted policies and championed the concept of ‘proportionate universalism.’ However, as Fisher and colleagues^3^ argue, it remains unclear how these approaches or a combination of them can be deployed to improve health equity in diverse contexts. Their paper explores this question synthesizing evidence from four Australian policy implementation case-studies: (*i*) the national broadband network; (*ii*) the national primary healthcare policy (PHC); (*iii*) the national indigenous health policy Closing the Gap; and (*iv*) land use policy in Sydney. Each case-study involved a mixture of universal and targeting approaches. They used three criteria to assess equity of access – availability, affordability and acceptability and looked at specific determinants of indigenous people’s health, including cultural safety.

 Two policies were considered universal: PHC and national broadband network. However, in both the baseline provision was found to restrict equity of access. For example, the episodic medical care baseline in PHC is a poor fit for people with chronic or non-communicable conditions (both more prevalent amongst lower socio-economic groups and indigenous people), services have low levels of cultural competency in terms of racist attitudes and practices and are better quality and more readily available in more advantaged areas. The National Broadband Network’s initial high-performing fibre-optics baseline had potential to be equitable across all three dimensions. However, changes introduced by a new government meant higher prices and lower performing technologies being used in more disadvantaged areas. Only one proportionate-universal service was identified: the new primary healthcare networks, responsible for population health planning with funding weighted according to population size plus rurality and socioeconomic factors – both proxy measures of disadvantage. However, it was too soon to assess how equity sensitive their implementation was.

 The research identified three types of targeted approaches across the case-studies. Publicly funded dental care for those who cannot afford private health insurance or out-of-pocket payments was judged to be residualist targeting. This service, tightly targeted at very specific groups and poorly resourced, is associated with significant inequalities in access and dental health outcomes between those with and without private health insurance. The other two types of targeting were services provided ‘within’ universal provision (eg, funding to encourage general practitioners to operate in underserved areas) and those provided alongside but separate from universal services (funding for Aboriginal Community Controlled Health Organisations - ACCHOs). A range of problems with all targeted services emerged from the relationship between regulatory agencies and service providers. These included: narrow targets that resulted in a poor ‘fit’ with local need; top-down performance management restricting flexibility; and insecure short-term funding. One ACCHO had 90 different targeted funding lines, each with different reporting requirements. Finally, the flexibility of devolved governance structures in all policy domains was evident in the way resources could be tailored to local conditions, but the benefits for equity of access could be limited or prevented by the exercise of control by central agencies.

 Fisher and colleagues^3^ conclude that their findings point to the continued importance of universal publicly funded services if social and health inequalities are to be reduced, but highlight the need for implementation practices to give equal weight to all dimensions of equity and for more extensive baseline provision to reduce the need for targeting. Additionally, however, they argue that at least in the Australian case “a mix of universal, proportionate and targeted implementation structures, including devolved governance structures, will be best suited to achieve equity in affordability, availability *and *acceptability, and therefore be best-placed to support health equity.”

 There are several reasons why this latter conclusion might be too hasty. First, their analysis could not take account of the “*cohesion dividend*” associated with universalism. In an interim report on the case for an international universalist welfare state and a global taxation system to support it Townsend^4^ argued that: “*The task is not just to re-introduce a successful historical model….It is to re-shape that model to meet new problems as well as problems that have been familiar for generations. The strength of a universalistic approach...is in building coalitions between groups in society.... Social security systems have created cross-cutting and three generational social identities and have moderated multiple forms of discrimination. Shrewdly interpreted, universalism can encompass rights by gender, race, ethnicity, age and disability*.”

 Second, universal approaches are less likely to exacerbate the shame and stigma which Sen^5^ argues is “at the absolutist core” of poverty and inequality. As long ago as 1968, Titmuss^6^ identified the difficulties of developing socially acceptable selective services that eliminate or significantly reduce stigma as central to the debate about the relative merits of universal versus targeted approaches. Research continues to show how ‘stigma’ operates in targeted approaches to service provision, damaging people’s health and reducing uptake, for example, in school based mental health services and mothers receiving public assistance benefits.^7,8^ The often-unintentional production of stigma in services and professional practices, works to discipline and divide social groups, determining who is viewed as deserving and underserving of public support. These processes reinforce the view amongst some public officials that inequalities are personal troubles not public issues.^9^ People do of course resist the imposition of stigma^10^ but it will not be eradicated from services by the further spread of targeting.

 Fisher and colleagues^3^ highlight the potential social dividend associated with some forms of targeting. For example, the ACCHOs operated as “vehicles for culturally relevant, strength-based strategies, supporting community empowerment and self-determination” and addressing upstream social determinants of health inequalities. However, they also note that ACCOHs are often run on the basis of goodwill or limited, insecure funding and lack consistent structural support and resources. Like other research this points to the need for fundamental change in how community-controlled targeted approaches are governed and implemented. For example, our study of a major English community empowerment programme identified significant benefits but also found these were unequally distributed within and across the communities and places involved.^11,12^

 Finally, what of proportionate universalism? Many targeted services are place-based but most disadvantaged people do not live in disadvantaged neighbourhoods/places. In Wales, for example, data for 2014 showed that if policies targeted action in the fifth most disadvantaged neighbourhoods, they would reach only around 37.5% of disadvantaged people. They might improve living conditions in these places but they would not impact on population level social and health inequalities. It was for this reason that Marmot recommended proportionate universalism. As he said in Fair Society Healthy Lives^13^ “Focusing solely on the most disadvantaged will not reduce the health gradient, and *will only tackle a small part of the problem….*To reduce the steepness of the social gradient in health, actions *must be universal*, but with a scale and intensity that is proportionate to the level of disadvantage.”

 The example of proportionate universalism in Fisher et al^3^ was the proportional funding of primary care networks. Similar ‘equity’ oriented resource allocation models are being developed at a sub-national level in the United Kingdom and they have potential to deliver more equitable access to services, but it is too early to assess the impact. More generally, proportionate universalism has been operationalised in very different ways. In Scotland, for example, it is defined as the resourcing and delivering of universal services that are able to respond differently to the level of presenting need. More often, however, resources are directed at more intensive services targeted at disadvantaged groups within drastically ‘hollowed out’ universal provision or no universal provision at all. This is partly because of reduced public sector funding but it is also because whilst a great idea in theory proportionate universalism is hard to achieve. It requires knowledge of how ‘need’ is distributed across the whole population, that is often not available and *place* is not a good proxy. Recently there have been important innovations in analytical methods that allow the distribution of need across whole populations to be more accurately mapped, for example, Multilevel Analysis of Individual Heterogeneity and Discriminatory Accuracy,^14^ which can also inform policy decisions relating to the potential for targeted approaches to effectively meet need. However, data availability may limit the potential of these sophisticated analytical approaches and even with accurate identification the spread of need across a population may mean that targeting will be challenging. Targeting, even in the context of proportionate universalism, therefore remains a problem practically for real life policy and program development. Additionally, the major problem of stigma limiting uptake of targeted services remains.

 For these reasons, we suggest an approach we call Equity Sensitive Universalism (ESU). This approach would arguably be easier to implement, albeit it requires progressive taxation systems, because the focus would be on achieving proportionate outcomes with equally provided resources, rather than targeted proportionate inputs. Universal child benefit, abolished in 2010 in the United Kingdom, was an ESU. It was available for all children, simple to deliver, efficient (with a progressive tax system to claim back the money not needed by affluent parents), non-stigmatising so uptake was comprehensive and therefore, at least in the United Kingdom, helped reduce child poverty. Non-shaming discipline strategies being implemented in Welsh schools, are also an ESU approach. All children will benefit but these will be greater for already traumatised children. Other examples of ESU policies being implemented in some countries include: the living wage, minimum unit pricing of alcohol, fluoridation, school breakfast clubs and early years services. A broader ESU strategy is described by Coote and Percy^15^ in their book on universal basic services.

 There is a strong case for targeting resources at local community-controlled initiatives that produce a social dividend in terms of self-determination and empowerment for marginalised and racialised groups, as is the case with the ACCHOs or where a group is readily identified and can co-design non-stigmatising services. But advocates for social and health justice should resist dominant arguments that universal approaches cannot adequately address social and health inequalities, that they are inefficient and/or create or exacerbate inequalities. Equity sensitive universal approaches can reduce inequalities: the problem is that too many governments are not willing to make the case for the progressive taxation these systems require and too few people are directing their imagination and energy at advocating for ESU to deliver social and health justice.

## Ethical issues

 Not applicable.

## Competing interests

 Authors declare that they have no competing interests.

## Authors’ contributions

 All authors contributed to drafting the paper led by RM and drawing on the work on ESU developed by CP and JP.

## Funding

 RM and JP were paid by their employers. CP made an in-kind contribution.
